# Comparison of the Efficacy of Three Loading Doses of Intravitreal Injection of Conbercept with Injection Combined with PDT for the Treatment of PCV

**DOI:** 10.1155/2020/2428348

**Published:** 2020-04-23

**Authors:** Fengjiao Li, Aihua Ma, Bojun Zhao

**Affiliations:** ^1^Department of Ophthalmology, Shandong Provincial Hospital Affiliated to Shandong University, Jinan, China; ^2^Department of Pediatrics, Shandong Provincial Hospital Affiliated to Shandong First Medical University, Jinan, China

## Abstract

**Purpose:**

To compare the efficacy between initial 3-monthly intravitreal conbercept monotherapy and combination intravitreal conbercept with photodynamic therapy (PDT) for polypoidal choroidal vasculopathy (PCV).

**Methods:**

This is a retrospective, comparative study which involved 65 PCV eyes of 65 patients. According to the therapeutic regimen, the PCV patients were divided into two groups: 32 eyes with naive PCV received a PDT after the first intravitreal injection of conbercept (IVC) followed by pro re nata (prn) retreatment (combination group), and 33 eyes with naïve PCV received 3-monthly IVC monotherapy followed by prn regimen (IVC monotherapy group). All patients completed at least 6 months of monthly follow-up.

**Results:**

At month 6, best-corrected visual acuity (BCVA) improved significantly (*P* < 0.05) in both groups compared with that at baseline; the mean changes of BCVA between the IVC monotherapy group and combination group have no significant difference (−0.22 ± 0.22 vs. −0.17 ± 0.22 LogMAR, *P* = 0.38). The central retinal thickness (CRT) decreased significantly in the two groups (*P* < 0.05), with no difference between the two groups (*P* = 0.24). The complete regression rate of polyps was 58.6% (17 out of 29 eyes) in the IVC monotherapy group and 80.65% (25 out of 31 eyes) in the combination group, respectively (*P* = 0.09, *χ*-squared test). The combination group required significantly fewer injections than the IVC monotherapy group (3.09 ± 0.89 vs. 3.67 ± 0.74, *P* = 0.006).

**Conclusion:**

Conbercept monotherapy significantly improved visual acuity and effectively regressed polyps during 6-month follow-up time in the treatment of PCV.

## 1. Introduction

PCV is defined as polypoidal lesions with or without branching vascular networks during early-phase indocyanine green angiography (ICGA) which is more prevalent in Asians as compared with whites [1]. The natural course of PCV may repeat hemorrhage and leakage which lead to vision deteriorate significantly [2, 3]. So the therapy for the treatment of PCV is necessary.

However, the optimal treatment for PCV has not been determined. Currently, the main treatment modalities include the combination of PDT with intravitreal injection of antivascular endothelial growth factor (VEGF) therapy and anti-VEGF monotherapy. The EVEREST-II study showed us that after 12 months, combination therapy of ranibizumab plus PDT was not only noninferior but also superior to ranibizumab monotherapy in terms of improvement of BCVA and regression of polyps. Combination therapy was recommended for the treatment of PCV [4]. The Fujisan study demonstrated that both initial and deferred PDT combined with intravitreal injection of ranibizumab to treat PCV achieved similar visual and anatomical improvements at 12 months. Initial PDT combination leads to significantly fewer additional treatments [5].

Various clinical trials have demonstrated the efficacy of anti-VEGF monotherapy in PCV, and currently, it has become one of the first-line treatments for PCV [6, 7]. Both ranibizumab and aflibercept have been used for the treatment of PCV. However, the low polyp resolution rate (ranging from 23% to 40%) [8, 9] for PCV patients has limited the clinical use of ranibizumab. Fortunately, aflibercept has been found more effective in polyp regression than ranibizumab [10].

Conbercept (KH902; Chengdu Kanghong Biotech Co., Ltd., Sichuan, China), a novel reagent of anti-VEGF, is a full humanized, soluble, VEGF receptor (VEGFR) protein. Compared to ranibizumab and bevacizumab, the most notable characteristic of conbercept is that it binds not only VEGF-A but also VEGF-B and placental growth factor (PIGF)—all with high affinity. The structure of conbercept is similar to aflibercept but they differ in that conbercept contains a fourth VEGFR-2 binding domain, which enhances the association rate of VEGF and prolongs its half-life in the vitreous [11].

Several studies have reported that IVC is safe and effective in the treatment of eyes with PCV [12–15]. One study exhibited that conbercept had greater effects on regressing polypoidal lesions compared with intravitreal ranibizumab injection in eyes with PCV [13]. In addition, intravitreal injection of conbercept using “3+prn” regimen had been reported to significantly improve visual acuity and anatomical outcomes in treatment-naive patients with PCV [14]. However, there have been no reports about the efficacy between IVC monotherapy and combination therapy in the treatment of PCV. Therefore, the objective of this study was to compare the efficacy between initial IVC monotherapy and combination of conbercept with PDT therapy for the treatment of PCV.

## 2. Methods

This was a retrospective, comparative study. Patients from Shandong Provincial Hospital were recruited between April 2017 and September 2019. The study was approved by the Medical Ethics Committee of Shandong Provincial Hospital, Shandong University, and adhered to the tenets of the Declaration of Helsinki. Study procedures, including the risks/benefits of intraocular injection therapy and PDT therapy, were explained to all participants before any treatments were initiated. All participants provided signed written informed consent.

### 2.1. Patients

A total of 65 eyes from 65 consecutive patients with symptomatic, treatment-naive PCV were included in the study. The inclusion criteria were [1] treatment-naive PCV characterized by the presence of polyps with or without branching vascular network (BVN) in the posterior pole of ICGA [2], observed subretinal fluid (SRF) or intraretinal fluid (IRF) under examination of optical coherence tomography (OCT) or leakage on the examination of fluorescein angiography (FA) [3], and completed at least 6 months of monthly follow-up after the first treatment. Exclusion criteria included (1) patients who had eyes with wet age-related macular degeneration (AMD) (2); previously underwent vitrectomy, laser photocoagulation, intravitreal triamcinolone injection, any intravitreal anti-VEGF injection, or PDT (3); and presence of other eye diseases such as glaucoma, diabetic retinopathy, and retinal angiomatous proliferation (RAP), which may affect visual acuity (VA).

The IVC monotherapy group was defined as patients who received three continuous monthly intravitreal injections of 0.5 mg conbercept followed by prn regimen; the combination group was defined as patients who initially received a single IVC followed by PDT within a week according to a standard protocol, followed by prn injections. Reinjections were considered if any intraretinal or subretinal fluid was observed on OCT and active polypoidal lesion was detected by OCT, fluorescein angiography, or ICGA. Patients were followed up monthly.

### 2.2. Examinations

At baseline and during the monthly follow-up visit, patients underwent comprehensive ophthalmologic examinations, including measurement of BCVA (Snellen), intraocular pressure (IOP), anterior segment slit-lamp examination, fundus examination and photography, and CRT by OCT (Cirrus OCT, Carl Zeiss Meditec, Dublin, CA, USA). All patients were generally scheduled to receive FA and ICGA (Heidelberg Engineering GmBH, Dossenheim, Germany) examination at 3 and 6 months after the initial treatment.

### 2.3. PDT

PDT was administered to the eyes in the combination group within one week after the first IVC. A standard dose (6 mg/m^2^) of verteporfin was administered according to the protocol. The greatest linear dimension (GLD) was defined as the area of polyps plus branching vascular neovascularization under ICGA examination, and the PDT treatment spot diameter was calculated as GLD plus 1000 *μ*m. A 689 nm laser system (Carl Zeiss, Dublin, CA, USA) was used to deliver 50 J/cm^2^ of energy to the treatment spot during an 83-second exposure.

### 2.4. The Intravitreal Injection Procedure

The injections were performed in the operating theater with a sharp 29-gauge needle. The needle was injected into the eye through the pars plana (3.5–4 mm posterior from the limbus); 0.05 ml of solution containing 0.5 mg of conbercept was injected.

### 2.5. Outcome Measurement

Outcome measurement included changes in BCVA (Snellen) and CRT, the rate of complete polyp regression, and injection numbers of IVC. Visual outcomes included the mean BCVA, the change of BCVA, and the proportion of VA improvement, stability, or deterioration compared to baseline. Snellen visual acuity was converted to LogMAR units for analysis. An improvement of ≥0.3 in LogMAR was defined as improvement of VA, an improvement of <0.3 in LogMAR was defined as stability of VA, and a decrease of >0.3 LogMAR was defined as deterioration of VA.

### 2.6. Statistical Analysis

All statistical analyses were performed using version 15.0 SPSS statistical software (SPSS, Inc., Chicago, IL. USA). Changes in BCVA and CRT before and after treatment were compared using paired-sample *t*-tests. Differences between treatment groups were compared using independent-sample *t*-tests. The *χ*-squared test was used to compare incidence rates between groups. *P* values < 0.05 were considered statistically significant.

## 3. Results

### 3.1. Baseline Characteristics

33 patients were included in the IVC monotherapy group, and 32 patients were included in the combination group. The baseline mean BCVA was 0.77 ± 0.29 LogMAR in the combination group and 0.68 ± 0.33 LogMAR in the IVC group. The baseline mean CRT was 394.22 ± 160.43 *μ*m in the combination group and 419.21 ± 169.85 *μ*m in the IVC group. Baseline characteristics included GLD and polyp type, and the rate of presence of large submacular hemorrhage did not differ significantly between the two groups.  Table 1 presents the baseline characteristics of subjects.

### 3.2. The Change of BCVA after Treatment in the Two Groups

**Figure 1:**
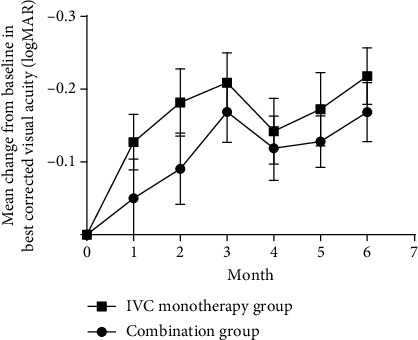
The mean changes of BCVA from baseline up to month 6 in the two study groups. Improvement in BCVA was maintained during 6 months in both groups, and no significant differences were observed between the two groups at any time point.

In the combination group, the baseline mean BCVA was 0.77 ± 0.29 LogMAR. The mean changes of BCVA from baseline to months 1, 3, and 6 were −0.05 ± 0.30, −0.17 ± 0.23, and −0.17 ± 0.22 LogMAR. Significant improvements in vision were observed at months 3 and 6 compared with baseline values: *P* < 0.001, respectively (Figure 1). Figure 1 illustrated mean changes of BCVA from baseline up to month 6 in the combination group. At month 6, the mean BCVA improved in 14 out of 32 (43.8%) and stabilized in 18 out of 32 (56.3%) patients. No patients showed deterioration in BCVA.

In the IVC monotherapy group, the baseline mean BCVA was 0.68 ± 0.33 LogMAR. The mean changes of BCVA from baseline to months 1, 3, and 6 were −0.13 ± 0.22, −0.21 ± 0.24, and −0.22 ± 0.22 LogMAR. Significant improvements in vision were observed at months 1, 3, and 6 compared with baseline values (*P* = 0.002, 0.000, and 0.002) (Figure 1). Figure 1 illustrated mean changes of BCVA from baseline up to month 6 in the IVC group. At month 6, the mean BCVA was improved in 17 out of 33 (51.5%) and stabilized in 16 out of 33 (48.5%) patients. No patient showed deterioration of BCVA.

In the comparison of the two groups, there was no statistically significant difference in terms of the mean changes of BCVA in every visiting time during 6 months of follow-up (*P* = 0.24, 0.18, 0.49, 0.71, 0.47, and 0.38, respectively) (Figure 1). No significant difference was found in the proportions of the changes of BCVA from baseline to 6 months (*P* = 0.62) between the two groups.

### 3.3. The Change of CRT after Treatment in the Two Groups

**Figure 2:**
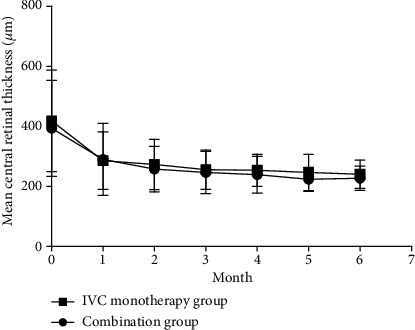
The mean CRT during 6 months in the two study groups. Reductions in the CRT were maintained over 6 months after treatment in the two groups, and no significant differences were observed between the two groups at any time point during the 6-month study period.

In the combination group, the mean CRT at baseline was 394.22 ± 160.43 *μ*m; this value was decreased significantly to 301.63 ± 108.6 *μ*m, 250.44 ± 71.99 *μ*m, and 227.81 ± 40.73 *μ*m at month 1,3,6, respectively (*P* < 0.01, respectively) (Figure 2). Figure 2 illustrated the CRT at baseline and each follow-up time point until month 6 in the combination group.

In the IVC group, the baseline mean CRT was 419.21 ± 169.85 *μ*m; this value was decreased significantly to 286.06 ± 96.52 *μ*m, 256.39 ± 65.54 *μ*m, and 240.97 ± 47.57 *μ*m at months 1, 3, and 6, respectively (*P* < 0.01, respectively) (Figure 2). Figure 2 illustrated the CRT at baseline and each follow-up time point until month 6 in the IVC group.

In the comparison of the two groups, there was no significant difference in terms of CRT at month 1–6 follow-up visit (*P* > 0.05), respectively (Figure 2).

### 3.4. Polyp Regression

60 eyes (92.3% of all subjects) received the FA and ICGA examinations at month 3 and 6 visits. At month 3 visit, complete regression of polypoidal vascular lesions was observed in 14 out of 29 eyes (48.28%) in the IVC group and in 24 out of 31 eyes (77.42%) in the combination group, respectively (*P* = 0.03, *χ*-squared test). At the month 6 visit, complete regression was observed in 17 (58.6%) of 29 eyes in the IVC group and in 25 (80.65%) of 31 eyes in the combination group, respectively (*P* = 0.09, *χ*-squared test).

### 3.5. IVC Numbers

The mean number of IVC during 6 months was 3.67 ± 0.74 in the IVC monotherapy and 3.09 ± 0.89 in the combination group. In summary, the combination group required significantly fewer treatments than the IVC group (*P* = 0.006).

### 3.6. Safety

No serious complications (e.g., intraocular inflammation, cataract, retinal detachment, retinal tears, and vitreous hemorrhage) occurred in either subgroup following the injections.

## 4. Discussion

This study compared initial treatment between IVC monotherapy and combination therapy for PCV during 6-month follow-up. To the best of our knowledge, this is the first report to compare the treatment efficacy between 3+prn IVC monotherapy and combination therapy for treatment-naïve eyes with PCV. Our result demonstrated that 3+prn IVC monotherapy achieved similar results in improvement of visual acuity and decrease of CRT as that of combination therapy. We also found that the rates of polyp regression had no statistical difference between the two groups. However, the total numbers of IVC in the combination group were significant lower than those in the IVC monotherapy group.

The most important goal for the treatment PCV should be improvement of vision [16]. Anti-VEGF treatment has been widely proven to be effective. In our study, at month 6, the mean BCVA improved by 0.22 LogMAR units in the IVC group; 51.5% eyes had an improvement of ≥0.3 in LogMAR visual acuity. Several studies showed the efficiency of “3+prn” IVC regimen in the treatment of PCV patients. Huang et al. demonstrated that 3-monthly IVC therapy followed by a prn regimen improved the mean BCVA of PCV patients (0.16 LogMAR) at 6-month follow-up [13]. Peng et al. reported that IVC monotherapy by using the “3+prn” regimen achieved visual acuity improvement ≥ 0.3 (LogMAR) in 60.42% (29/48) of eyes [14]. Our result was similar to those previous reports.

When compared with the combination group, our results showed that conbercept monotherapy had no difference in improvement of VA and reduction of CRT. The structure of conbercept is similar with that of aflibercept. Comparison of aflibercept monotherapy and aflibercept combination with PDT had been reported [17, 18]. Takayama et al. reported that aflibercept monotherapy achieved similar visual acuity improvements and anatomical recovering as compared to combination therapy [17]. Kikushima et al. also reported that aflibercept monotherapy and combination therapy were equivalent in achieving visual gain after 12 months of treatment [18].

The secondary goal of PCV treatment was to achieve complete polyp closure. Although polyp regression does not necessarily indicate a good vision prognosis, neither is there clear evidence of a direct association between higher polyp closure rates and lower recurrences, the resolution of polyps was considered the end point of treatment in clinical practice. Although there are no head-to-head studies to compare directly among different anti-VEGF agents, there may be differences among different anti-VEGF agents in the regression of the polyps. A previous study demonstrated that aflibercept may have an advantage in the regression of the polyps than ranibizumab [10]. In our study, the rate of complete polyp regression of conbercept at the end of 6-month follow-up was about 58.6%. This is in consistent with a previous study which reported that the rate of conbercept regression of the polyps ranged from 43.75% to 78.6% [13–15, 19]. The AURORA study reported that complete regression of polyps was observed in 56.5% of patients in the 0.5 mg IVC group [15]. In our study, combination therapy was not superior to conbercept monotherapy in achieving complete polyp regression over 6months (80.65% vs. 58.6%, *P* = 0.09). The PLANET study which compared the efficacy and safety of aflibercept monotherapy versus aflibercept with PDT rescue therapy demonstrated that both treatment arms achieved similar polyp regression rates (38.9% vs. 44.8%, *P* = 0.32), which concluded there was no additional benefit by adding rescue PDT in polyp regression [7]. So both conbercept and aflibercept monotherapy had a high polyp regression rate. However, the mechanism for conbercept and aflibercept with the high polyp regression rate still remains to be investigated. When compared with ranibizumab, this may be due to the fact that both conbercept and aflibercept have high affinity not only for VEGF-A but also for placental growth factor and VEGF-B, whereas ranibizumab can only inhibit VEGF-A.

During the follow-up period, the incidence of IVC numbers in the present study was significantly higher in the IVC monotherapy group than that in the combination group. Nevertheless, the disadvantages of PDT include the need for special equipment and the high cost. Moreover, in a few cases, PDT may lead to subretinal hemorrhage, choroidal infarct, and RPE tear, which result in acute vision loss. These disadvantages and side effects of PDT may limit its clinical use.

The limitations of the current study include the short follow-up period and the small sample size. To further validate these conclusions, it will be necessary to perform a large-scale randomized and multicenter clinical trial.

## 5. Conclusion

Conbercept monotherapy significantly improved visual acuity and effectively regressed polyps at least within 6 months of treatment in eyes with naive PCV.

## Figures and Tables

**Table 1:** Baseline characteristics of subjects.

	Combination group	IVC monotherapy	*P* value
*n* (eyes)	32	33	—
Age (years)	66.97 ± 9.91	69.27 ± 6.44	0.27
Gender (male/female)	13/19	16/17	0.34
Baseline BCVA	0.77 ± 0.29	0.68 ± 0.33	0.22
Baseline CRT (*μ*m)	394.22 ± 160.43	419.21 ± 169.85	0.54
Greatest linear dimension (GLD) (*μ*m)	2688.16 ± 1143.79	2585.30 ± 1097.74	0.7
Polyp type (solitary/clustered)	9/23	6/27	0.38
Presence of large submacular hemorrhage (>4 disk areas)	4/28	6/27	0.73

Values were shown as means ± standard deviations. BCVA: best-corrected visual acuity; LogMAR: logarithm of the minimal angle resolution; CRT: central retinal thickness.

## Data Availability

We can provide the data if we have a reasonable request.
