# Corrigendum to “Comparison of the Efficacy of Three Loading Doses of Intravitreal Injection of Conbercept with Injection Combined with PDT for the Treatment of PCV”

**DOI:** 10.1155/2020/2423839

**Published:** 2020-08-26

**Authors:** Fengjiao Li, Aihua Ma, Bojun Zhao

**Affiliations:** ^1^Department of Ophthalmology, Shandong Provincial Hospital Affiliated to Shandong First Medical University, Jinan, Shandong 250021, China; ^2^Department of Ophthalmology, Shandong Provincial Hospital, Cheeloo College of Medicine, Shandong University, Jinan, Shandong 250021, China; ^3^Department of Pediatrics, Shandong Provincial Hospital Affiliated to Shandong First Medical University, Jinan, Shandong 250021, China; ^4^Department of Pediatrics, Shandong Provincial Hospital, Cheeloo College of Medicine, Shandong University, Jinan, Shandong 250021, China

In the article titled “Comparison of the Efficacy of Three Loading Doses of Intravitreal Injection of Conbercept with Injection Combined with PDT for the Treatment of PCV” [1], authors Dr. Fengjiao Li and Dr. Bojun Zhao were not affiliated to “Department of Ophthalmology, Shandong Provincial Hospital, Cheeloo College of Medicine, Shandong University, Jinan, Shandong, 250021, China,” and Dr. Aihua Ma was not affiliated to “Department of Pediatrics, Shandong Provincial Hospital, Cheeloo College of Medicine, Shandong University, Jinan, Shandong, 250021, China.”

The corrected list of affiliations is shown in the author information above.
